# What we learn from weed genetics

**DOI:** 10.1002/ajb2.70190

**Published:** 2026-04-08

**Authors:** Acer VanWallendael

**Affiliations:** ^1^ Department of Horticultural Sciences North Carolina State University Raleigh 27695‐7609 North Carolina USA; ^2^ Department of Crop & Soil Sciences North Carolina State University Raleigh 27695‐7620 North Carolina USA

**Keywords:** eccDNA, extrachromosomal, herbicide resistance, parasitic, pooled sequencing, population genetics, rapid evolution, weeds

Pest plants like agricultural weeds have always been useful systems for studying plant evolutionary ecology, because some of their traits evolve rapidly enough to be measured in reasonable timescales. In the past decade, genetic tools and resources previously only available for model species and crops have become accessible for weeds (defined here as ruderal nuisance plants *sensu* Baker, 1974). Many of these resources were initially developed to diagnose and understand the evolution of herbicide resistance, and along the way have facilitated the discovery of a fascinating array of genetic phenomena and unique adaptations to anthropogenic disturbance. Weeds, by definition, are the plant kingdom's proven experts at thriving under human influence. Geneticists are now probing the diversity present in weeds not only to solve pressing issues in agriculture and conservation, but also to use weed populations as natural experiments in evolutionary genetics.

## GENETIC PHENOMENA IN WEEDS

The first attempt to use weeds as genetic models came well before the rise to fame of *Arabidopsis* (Brassicaceae), when Gregor Mendel moved from researching peas to studying the genus *Hieracium* (Asteraceae), the hawkweeds. Mendel's research in hawkweeds did not match his findings in peas, since (as it was later discovered) hawkweeds reproduce by apomixis and are often polyploid. In the modern era, applied weed scientists and evolutionary geneticists have studied weeds to discover new ways to control them and to gain insights from their rapid adaptation. Here, I highlight three phenomena studied in weeds that may not be widely known.

### Herbicide‐resistant extrachromosomal circular DNA

Extrachromosomal circular DNA (eccDNA) is formed regularly in the process of transposable element (TE) recognition and repair processes, as well as through errors in chromosome replication (Zhang et al., 2023). The small circular pieces of DNA that resemble bacterial plasmids are not typically expressed and rarely inherited in offspring. In 2018, however, Koo et al. (2018) reported that resistance to the herbicide glyphosate in a population of palmer amaranth (*Amaranthus palmeri* S.Watson; Amaranthaceae) was expressed through many copies of a ~ 300 kb eccDNA fragment that included the glyphosate target gene. Since 2018, herbicide resistance‐related eccDNA has been found in additional species such as the grasses (Poaceae) *Lolium perenne* ssp. *multiflorum* (Lam.) Husnot and *Alopecurus myosuroides* Huds. (Fu et al., 2023; Koo et al., 2023). In 2025, researchers reported a population of *Am. palmeri* with a second herbicide resistance (HR) mechanism on its eccDNA, resistance to the herbicide glufosinate (Carvalho‐Moore et al., 2025). With improved reference genomes and tools to sequence and detect larger circularized DNA, efforts are ongoing to determine how prevalent this phenomenon is in weed populations, and to model its spread across regions. Understanding the expression and inheritance of eccDNA molecules has been useful for weed biologists, but has also captured the attention of synthetic biologists seeking novel ways to introduce new traits into plant genetic lines (Mohan et al., 2024). If traits can be introduced on stable, heritable eccDNAs, researchers will avoid the often‐problematic step of vector integration into nuclear DNA.

### Metabolic resistance to herbicides

All plants possess a degree of tolerance to many xenobiotics such as herbicides via metabolic detoxification pathways. Detoxification genes in the cytochrome P450 and glutathione S‐transferase families have been well‐described in insects, where they may have evolved to degrade plant‐produced toxins (Scott, 1999). In weeds, upregulation of detoxification genes in the same families is a common mechanism of non‐target site herbicide resistance (resistance that does not involve a change in the protein targeted by the herbicide) and is particularly challenging to manage since it can allow weeds to simultaneously evolve resistance to numerous herbicides. Previous research has identified compounds that can upregulate detoxification when applied to crops to prevent accidental herbicide damage (e.g., benoxacor in maize), and others that can downregulate detoxification in weeds to mitigate metabolic resistance (e.g., malathion). Despite years of research, however, the sheer diversity of cytochrome P450 genes in many weed species has made functional study challenging. Recent genomic work has focused on automating the detection and annotation of detoxification genes in newly released genomes (Rigon et al., 2025). A major question remains whether HR due to metabolic resistance more commonly arises from single genes or from changes in transcriptional regulation of multiple genes simultaneously, since both have been found across different weed species (Bai et al., 2018; Han et al., 2021). Bringing together genomic classification and functional descriptions of detoxification genes may lead to novel herbicides that are less likely to face metabolic resistance and may provide insight into how plants will adapt to future classes of anthropogenic environmental toxins.

### Horizontal gene transfer in parasitic weeds

As challenging as plant taxonomy can be at times, phylogeneticists have not typically needed to consider the headache of the bacterial world, where conjugation and transformation wreak havoc on sensible trees. However, researchers of parasitic plants may have it especially tough, as genetic exchanges resembling bacterial horizontal gene transfer have been found in at least five genera of parasitic plants: *Striga* (Orobanchaceae), *Orobanche* (Orobanchaceae), *Phelipanche* (Orobanchaceae), *Rafflesia* (Rafflesiaceae), and *Cuscuta* Convolvulaceae) (Clarke et al., 2019). *Striga* and *Cuscuta* include species that are often problematic weeds in their native ranges. Horizontal transfer might occur through the broad exchange of mRNA through haustorial links, some of which may be reverse‐transcribed and incorporated into the nuclear genome of either host or parasite (Yang et al., 2019). Gene transfer in weedy parasitic plants has so far not resulted in clear adaptive advantages. Although few direct applications of this phenomenon have been suggested, the possibility to send genes across species through *Cuscuta* wires is a tantalizing and frightening possibility.

## HORIZON SCAN

**Figure 1:**
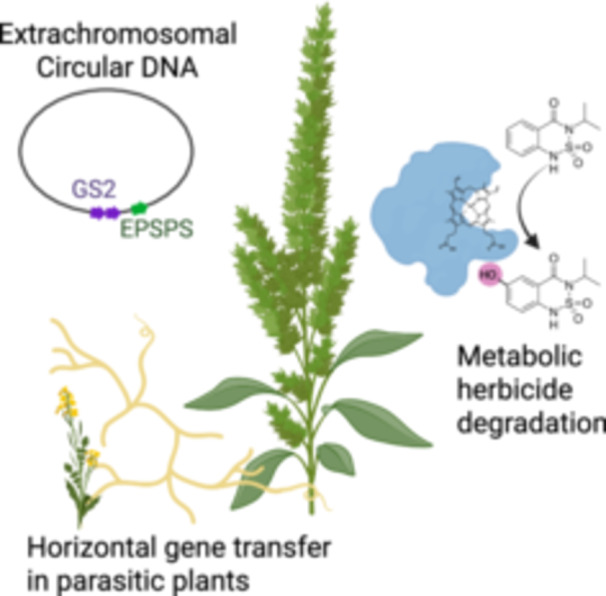
Notable genetic phenomena in weed biology (VanWallendael, 2026). *Upper left:* Some weeds have evolved the use of extrachromosomal circular DNA to replicate herbicide target enzymes, including both GS2 and EPSPS in *Amaranthus palmeri* (Carvalho‐Moore et al., 2025). *Right:* Metabolic enzymes can detoxify herbicides in weeds and crop species; here is shown P450‐mediated detoxification of bentazon discovered in rice (Dimaano and Iwakami, 2021). *Lower left:* Parasitic weeds such as *Cuscuta* spp. have been found to exchange genetic material with host species.

I have been fortunate enough to learn from some of the best long‐term experiments in evolution and ecology, from the Beal seed experiment (Telewski and Zeevaart, 2002) and Long‐Term Evolution Experiment (Lenski, 2004) at Michigan State University to Project Baseline at Fordham University (Etterson et al., 2016). These projects were deeply in my mind as I began a new lab studying weed genetics, wrestling with ideas to experimentally address larger questions in weed science, and plant evolution more broadly. The core challenge in designing experiments to study the factors that govern weed adaptation is that we almost never have a view of a weed population as it existed before evolving a new trait such as herbicide resistance or early flowering. We are attempting to change that through a multi‐year, multi‐species pooled sequencing project of agricultural weed species in the United States that we are calling PopuWeed. The idea is simple in concept, i.e., we collect leaves from numerous weed populations at set locations every year, then use pooled whole‐genome sequencing to identify shifts in allele frequencies over time and across space. The populations we use are a mix of weeds with different levels of existing herbicide resistance, and include selfers, outcrossers, monocots, and eudicots. The first two years of collections have already filled our new lab freezer with snapshots of around 20 populations of weeds as they existed in 2024 and 2025. With each passing year, we gain more power to address questions in rapid plant evolution and weed genetics. Does mating system govern the degree to which new traits come from mutations or gene flow? Is the fate of parallel novel mutations the same across different populations or in different years? How does the abiotic, biotic, and anthropogenic environment alter the trajectory of weed evolution? What role do eccDNA, metabolic resistance, and horizontal gene transfer (through *Cuscuta* or through viruses) play in producing genetic novelty? (Figure 1).

The world of plants faces constant anthropogenic pressure through land‐use and climate change. I believe that taking the time to learn from the species best adapted to thrive under hostile conditions will benefit plant biology and help protect biodiversity. With the PopuWeed project and similar exciting efforts from researchers around the world, we continue to uncover unexpected strategies that make us constantly rethink what we know about plant genomes.
